# An amiloride derivative is active against the F_1_F_o_-ATP synthase and cytochrome *bd* oxidase of *Mycobacterium tuberculosis*

**DOI:** 10.1038/s42003-022-03110-8

**Published:** 2022-02-24

**Authors:** Kiel Hards, Chen-Yi Cheung, Natalie Waller, Cara Adolph, Laura Keighley, Zhi Shean Tee, Liam K. Harold, Ayana Menorca, Richard S. Bujaroski, Benjamin J. Buckley, Joel D. A. Tyndall, Matthew B. McNeil, Kyu Y. Rhee, Helen K. Opel-Reading, Kurt Krause, Laura Preiss, Julian D. Langer, Thomas Meier, Erik J. Hasenoehrl, Michael Berney, Michael J. Kelso, Gregory M. Cook

**Affiliations:** 1 Department of Microbiology and Immunology, University of Otago, Dunedin, New Zealand; 2 Maurice Wilkins Centre for Molecular Biodiscovery, The University of Otago, Dunedin, New Zealand; 3 Molecular Horizons and School of Chemistry and Molecular Bioscience, University of Wollongong, Wollongong, Australia; 4 Illawarra Health and Medical Research Institute, Wollongong, Australia; 5 School of Pharmacy, University of Otago, Dunedin, New Zealand; 6 Weill Department of Medicine, Weill Cornell Medical College, New York, NY USA; 7 Department of Biochemistry, University of Otago, Dunedin, New Zealand; 8 Department of Structural Biology, Max-Planck Institute of Biophysics, Frankfurt am Main, Germany; 9 Octapharma Biopharmaceuticals GmbH, Heidelberg, Germany; 10 Department of Molecular Membrane Biology, Max-Planck Institute of Biophysics, Frankfurt am Main, Germany; 11 Department of Life Sciences, Imperial College London, London, UK; 12 Private University in the Principality of Liechtenstein, Triesen, Liechtenstein; 13 Department of Microbiology and Immunology, Albert Einstein College of Medicine, New York, NY USA

**Keywords:** Pathogens, Drug development

## Abstract

Increasing antimicrobial resistance compels the search for next-generation inhibitors with differing or multiple molecular targets. In this regard, energy conservation in *Mycobacterium tuberculosis* has been clinically validated as a promising new drug target for combatting drug-resistant strains of *M. tuberculosis*. Here, we show that HM2-16F, a 6-substituted derivative of the FDA-approved drug amiloride, is an anti-tubercular inhibitor with bactericidal properties comparable to the FDA-approved drug bedaquiline (BDQ; Sirturo^®^) and inhibits the growth of bedaquiline-resistant mutants. We show that HM2-16F weakly inhibits the F_1_F_o_-ATP synthase, depletes ATP, and affects the entry of acetyl-CoA into the Krebs cycle. HM2-16F synergizes with the cytochrome *bcc-aa*_*3*_ oxidase inhibitor Q203 (Telacebec) and co-administration with Q203 sterilizes in vitro cultures in 14 days. Synergy with Q203 occurs via direct inhibition of the cytochrome *bd* oxidase by HM2-16F. This study shows that amiloride derivatives represent a promising discovery platform for targeting energy generation in drug-resistant tuberculosis.

## Abstract (web summary)

Derivatives of the FDA-approved drug, amiloride, can eliminate drug-resistant *Mycobacterium tuberculosis* in vitro by interfering with bacterial energy conservation.

## Introduction

Tuberculosis (TB) is a leading cause of mortality globally, with over one million deaths annually^1^. The emergence of multidrug-resistant (MDR), extensively drug-resistant (XDR) and totally drug-resistant (TDR) strains of *Mycobacterium tuberculosis* are resulting in extremely limited treatment options^2^. Current drugs for treating drug-resistant TB disease are slow-acting and treating drug-sensitive strains requires the use of up to four drugs for at least six months^3^. At present, MDR-TB is treated with a combination of eight to ten drugs lasting 18-24 months^4^. Bedaquiline (BDQ; Sirturo^®^) was approved by the US FDA in 2012 for the treatment of adults with pulmonary MDR-TB^5,6^. BDQ targets the energy-generating machinery (F_1_F_o_-ATP synthase) of *M. tuberculosis*^7,8^ marking energy generation a compelling target space for antimicrobial drug development^9^.

BDQ is generally bactericidal and can kill highly drug-resistant mycobacterial species and dormant bacilli^10,11^. It acts quickly compared to most TB drugs, but still requires many weeks of therapy and BDQ-resistance has been reported, including in treatment-naïve populations^12,13^. BDQ binds to the *c*-subunit rotor in the membrane-embedded part of the F_1_F_o_-ATP synthase^8^, inhibiting the enzyme by tightly binding to the *a–c* subunit interface^14^ and decreasing intracellular ATP levels^15,16^. BDQ also dissipates the ΔpH component of the proton-motive force in mycobacteria^17,18^. This depends on the target-based accumulation of BDQ and leads to an uncoupled microenvironment around the F_1_F_o_-ATP synthase^18^. Targeting multiple mechanisms within energy generation thus appears to be key for developing efficacious anti-tubercular compounds^19^.

Targeting respiratory complexes earlier in the process of oxidative phosphorylation has begun to provide an understanding on how multi-targeting respiratory therapies can be designed. Telacebec^®^ (Q203;^20^), has been developed as an inhibitor of *M. tuberculosis* cytochrome *bcc*:*aa*_3_ terminal oxidase, one of two terminal oxidases that catalyze the terminal reduction of oxygen during cellular respiration^20^. Although bacteriostatic on its own, Q203 is rapidly and potently bactericidal when a secondary terminal oxidase, cytochrome *bd*, is deleted^21^. We recently demonstrated that the cytochrome *bd* inhibitor ND-011992 strongly synergizes with Q203 and the combination can kill antibiotic-tolerant hypoxic *M. tuberculosis*^22^. However, the less-than-optimal pharmacokinetic properties of ND-011992 make it unsuitable for development and alternative chemical scaffolds targeting cytochrome *bd* are required. Notably, Q203 itself shows no synergy with BDQ^23^. It is unknown whether compounds can be developed to exploit the best properties of both BDQ and terminal oxidase inhibition.

Several studies have identified new scaffolds for developing next-generation F_1_F_o_-ATP synthase inhibitors, such as squaramides^24^ and quinoline arylsulfonamides^25^. However, these compounds have limited pre-existing data regarding their clinical safety and efficacy. In contrast, few studies have identified inhibitors of cytochrome *bd*. We hypothesized that bioisosterism between BDQ and amiloride could be exploited to identify amiloride-based inhibitors of the mycobacterial F_1_F_o-_ATP synthase and thus mycobacterial growth. Amilorides as a drug class were originally identified as potassium-sparing diuretics in 1967^26^. The parent drug, amiloride, is still in use today as tablets (Midamor^TM^). In this study, we investigated the ability of a previously synthesized 6-substituted amiloride derivative, HM2-16F^27^, to function as an antimycobacterial F_1_F_o_-ATP synthase inhibitor. HM2-16F has previously been optimized to eliminate the diuretic activity of amiloride while having promising pharmacological properties for the treatment of cancers driven by the urokinase-type plasminogen activator^27^. Here, we found that HM2-16F shows comparable killing kinetics to BDQ and displays a different resistance profile at the level of the F_1_F_o_-ATP synthase *c*-ring, although its direct inhibition of the F_1_F_o_-ATP synthase was found to be comparatively poor. We show that HM2-16F possesses activity as a cytochrome *bd* inhibitor and potently synergizes with Q203, comparable to ND-011992^22^. We propose that amilorides represent a promising scaffold for antitubercular drug development, particularly in combination with Q203. HM2-16F may be a promising starting point for developing compounds that target both the F_1_F_o_-ATP synthase and terminal oxidases of *M. tuberculosis*.

## Results

### Amiloride derivatives are selectively active against the growth and survival of mycobacterial species

**Fig. 1:**
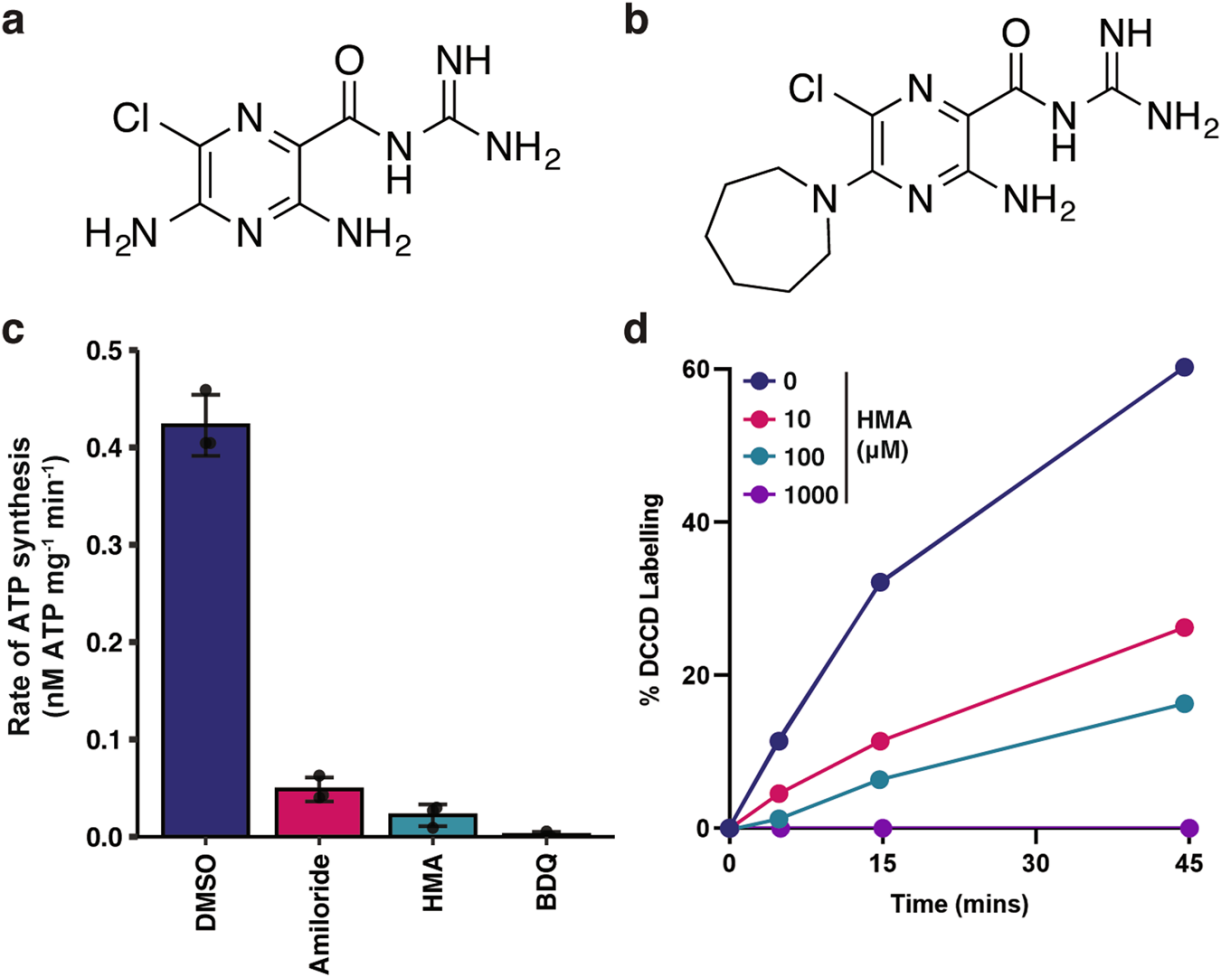
Inhibition of the mycobacterial F_1_F_o_-ATP synthase by amiloride and hexamethylene amiloride. **a** Structure of amiloride and **b** 5-(N,N-hexamethylene)amiloride (HMA). **c** ATP synthesis activity of NADH-energized *M. smegmatis* inverted membrane vesicles (IMV), treated with either 64 μM of amiloride, 64 μM of HMA, or 2 μM of BDQ as indicated. Error bars represent standard deviation from triplicate biologically independent measurements. **d** Time course of DCCD labelling in purified *M. phlei* c-rings in the presence of the indicated amounts of HMA. Data are representative of duplicate measurements.

We hypothesized that bioisosterism between BDQ and amiloride could be exploited to identify amiloride-based inhibitors of the mycobacterial F_1_F_o-_ATP synthase and thus mycobacterial growth. Amiloride (Fig. 1a) and the more hydrophobic 5-substituted derivatives 5-(*N*-ethyl-*N-*isopropyl)-amiloride (EIPA) and 5-(*N*,*N*-hexamethylene)amiloride (HMA, Fig. 1b) were tested for their ability to inhibit the growth of *M. tuberculosis* and a selection of bacterial pathogens (Table 1). Amiloride and EIPA were able to inhibit the growth of *M. tuberculosis* at 64–256 μM while a further drop in the minimal inhibitory concentration (MIC) was noted for HMA (32 μM, Table 1). Bedaquiline and isoniazid were included as positive controls in these experiments and exhibited the expected MIC values for these inhibitors (Table 1). Both amiloride and HMA were able to inhibit ATP synthesis in inverted membrane vesicles (IMVs) of *M. smegmatis* at concentrations comparable to their MIC (Fig. 1c and Table 1). In support of this, HMA was able to outcompete the canonical *c*-ring covalent inhibitor DCCD for inhibition of the *M. phlei* F_1_F_o_-ATP synthase c-subunit (Fig. 1d). The compounds were poorly active against the growth of other bacterial pathogens, suggesting some selectivity towards mycobacteria (Table 1).

**Table 1:** Minimum inhibitory concentrations (MIC, μM) of amiloride and derivatives against *M. tuberculosis* and selected bacterial pathogens.

Compound	*Mycobacterium tuberculosis* H37Rv	*Escherichia coli*	*Staphylococcus aureus*	*Enterococcus faecalis*
Amiloride	256	>512	>512	>512
HMA	32	128	128	512
EIPA	64	>512	>512	512
HM2-16F	4	256	>256	256
Bedaquiline	0.2	ND	ND	ND
Isoniazid	0.2	ND	ND	ND

All MIC values are reported in micromolar final concentration. ND = not determined as inactive against these bacteria. All strains are listed in Supplementary Table 1.

**Fig. 2:**
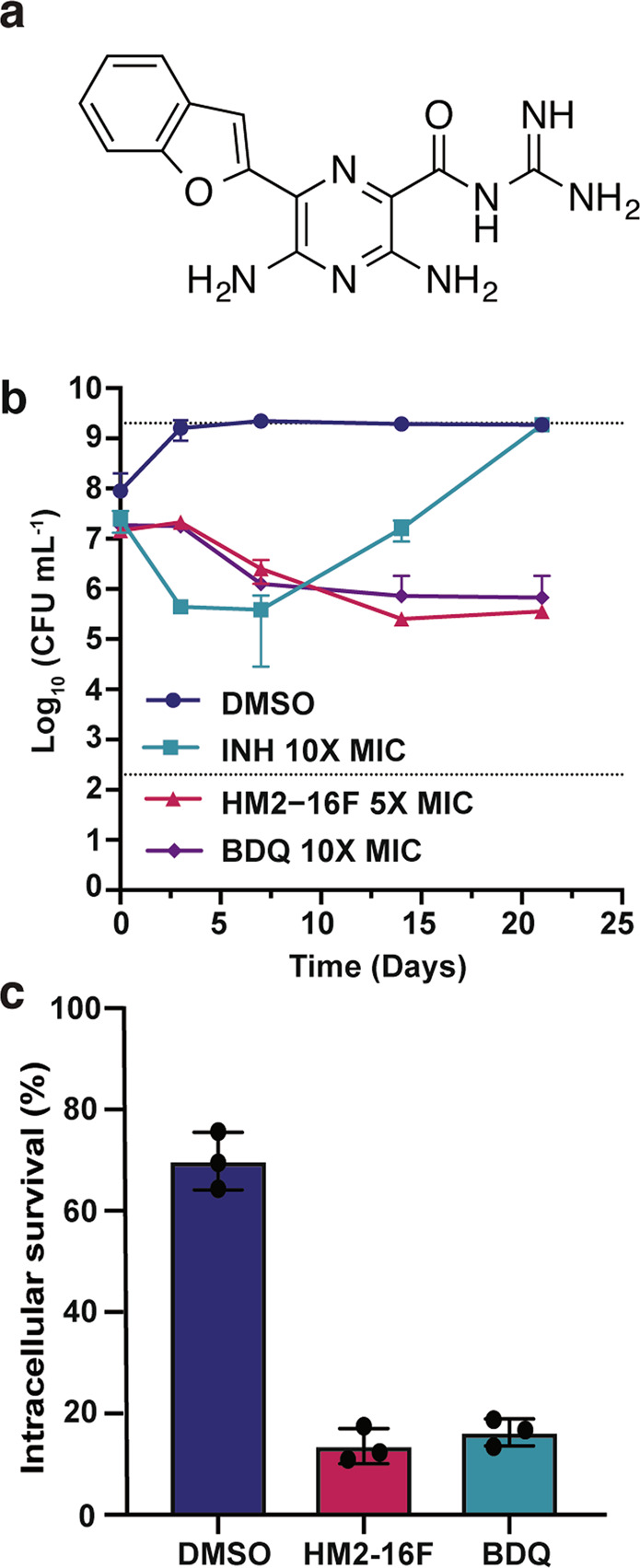
HM2-16F is bactericidal towards mycobacteria in vitro and in macrophages. **a** Structure of HM2-16F. **b** Survival of *M. tuberculosis* after treatment with: isoniazid (INH 2 μM, 10× MIC), HM2-16F (20 μM, 5× MIC) and bedaquiline (BDQ 2 μM, 10X MIC). **c** Survival of *M. bovis* BCG in human THP-1 macrophages after treatment with 5 μM HM2-16F and BDQ. Error bars indicate standard deviation from three biological independent experiments.

Amiloride is an approved potassium-sparing diuretic with a low maximum daily dose (~20 mg/day)^26^; an activity that would be incompatible with long-term treatment of tuberculosis patients. We previously reported on a 6-substituted derivative (HM2-16 F; Fig. 2a) that shows no inhibition of the human epithelial sodium channel (ENaC) in vitro and minimal diuretic or antikaliuretic properties in rats^27^. HM2-16F was found to be more potent growth inhibitor against *M. tuberculosis*, showing a 64- and 8-fold reduction in MIC compared to amiloride and HMA, respectively (Table 1). HM2-16F was also highly selective for *M. tuberculosis*, minimally inhibiting the growth of *Escherichia coli, Staphylococcus aureus* or *Enterococcus faecalis* (Table 1). HM2-16F was bactericidal towards *M. tuberculosis* at 5X MIC (20 μM), achieving 1.5 log_10_ CFU mL^-1^ killing of cells over 15 days (Fig. 2b). The efficacy and kinetics of killing were comparable to BDQ at 10X MIC (2 μM, Fig. 2b), and did not show generation of persisters/resisters in comparison to treatment with isoniazid at 10X MIC (2 μM, Fig. 2b). HM2-16F was also able to prevent the survival of *M. bovis* in THP-1 macrophages in a manner comparable to BDQ (Fig. 2c). Under hypoxia (non-replicating cultures), both HM2-16F and BDQ had no bactericidal activity over the time course of 30 days (Supplementary Fig. 1).

### Interaction of HM2-16F with the F_1_F_o_-ATP synthase

**Fig. 3:**
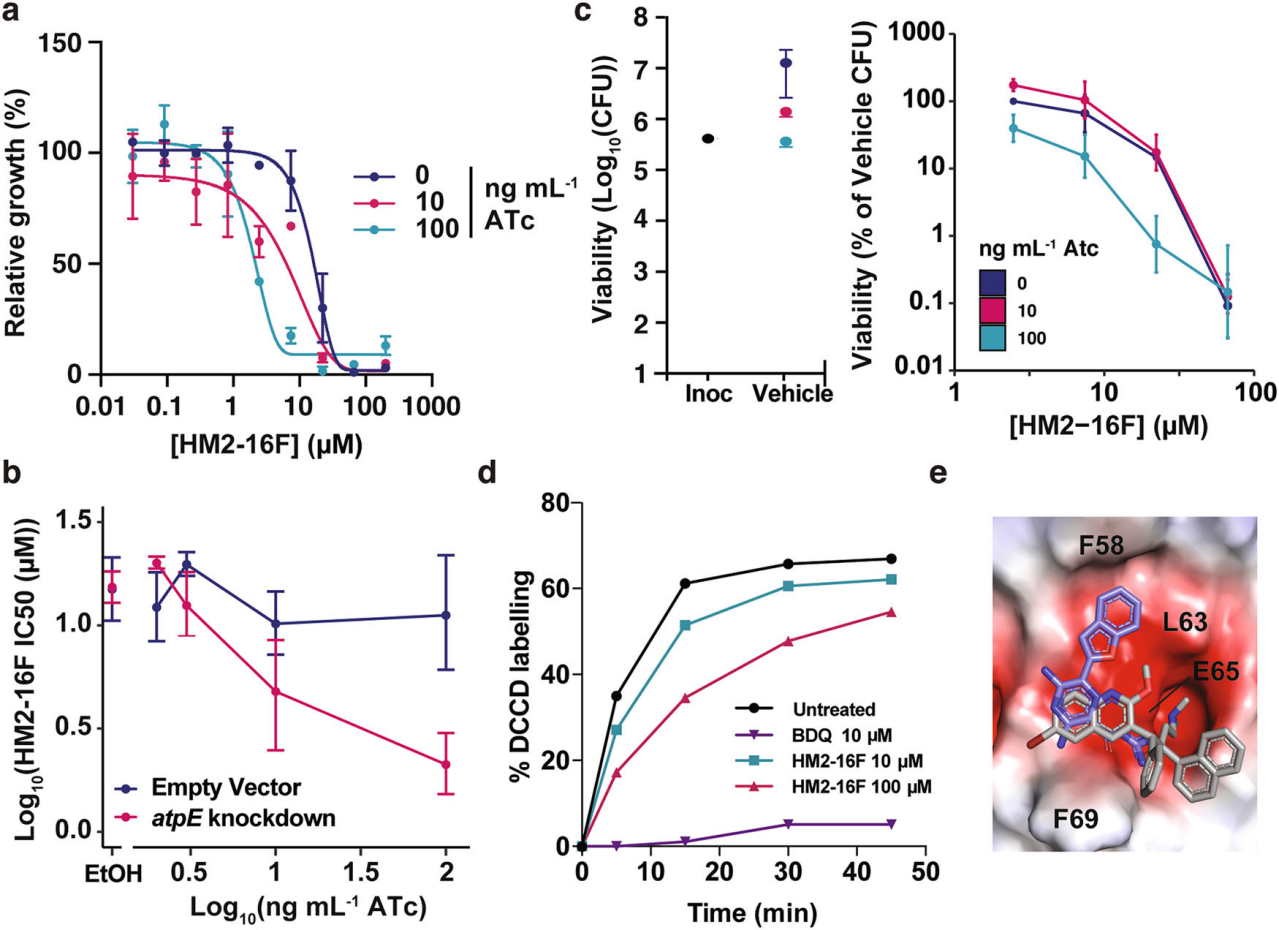
Interactions of HM2-16F with the mycobacterial F_1_F_o_-ATP synthase. **a** Knockdown of the ATP synthase operon (sgRNA targeting *atpB* – pCi74) was induced with the indicated amounts of ATc and the growth relative to the vehicle control was determined. Error bars represent standard deviation from three biological independent experiments. **b** Growth after 10 days and IC_50_ of HM2-16F as a function of F_1_F_o_-ATP synthase knockdown (sgRNA targeting *atpB* – pCi74). Error bars represent 95% confidence interval. **c** Viability of *M. tuberculosis* after 10 days was determined in an analogous experiment to panel **a** at 0, 10 and 100 ng mL^−^^1^. **d** Time course of DCCD labelling in purified *M. phlei* c-rings in the presence of the indicated amounts of compounds. Data are representative of duplicate measurements. **e** HM2-16F (purple carbons) docked into the BDQ binding site of mycobacterial F_1_F_o_-ATP synthase *c*-ring (PDB ID: 4V1F). The high-resolution c-ring-BDQ structure is shown in grey as a reference. The protein is shown as an electrostatic potential surface (red – electronegative, white – neutral, blue – electropositive; generated in PyMOL). Amino acid positions refer to the *M. phlei* protein. The top three ranked poses are shown in Supplementary Figure 5.

To investigate whether HM2-16F targets the mycobacterial ATP synthase at the whole-cell level, we used CRISPR interference (CRISPRi) to generate a transcriptional knockdown of the *a*-subunit of the F_1_F_o_-ATP synthase *(atpB* gene) in *M. tuberculosis* mc^2^6230. This *atpB* knockdown was previously shown to reduce transcription by ~80 fold and increase sensitivity to BDQ, demonstrating its utility for validating inhibitors of the ATP synthase^28^. Addition of increasing concentrations of ATc, which induces CRISPRi activation and *atpB* transcriptional repression^28^, reduced the MIC of HM2-16F by ~7 fold (MIC = 34 and 4 μM with 0 and 100 ng/ml ATc respectively), while a non-targeting single-guide RNA (sgRNA) did not affect the MIC (Figs. 3a, b and Supplementary Figs. 2 and 3). Viability assays showed a similar trend for killing of cells in an equivalent experiment (Fig. 3c). This suggests that the transcriptional repression of the mycobacterial F_1_F_o_-ATP synthase reduced the concentration of HM2-16F required to inhibit *M. tuberculosis*. HM2-16F (10 μM) was only able to weakly outcompete the *c*-ring inhibitor DCCD for binding at the *M. phlei c*-ring (Fig. 3d), compared to the potent inhibition displayed by BDQ (10 μM, 50X MIC), suggesting that HM2-16F results in only modest binding/inhibition of the F_1_F_o_-ATP synthase.

**Fig. 4:**
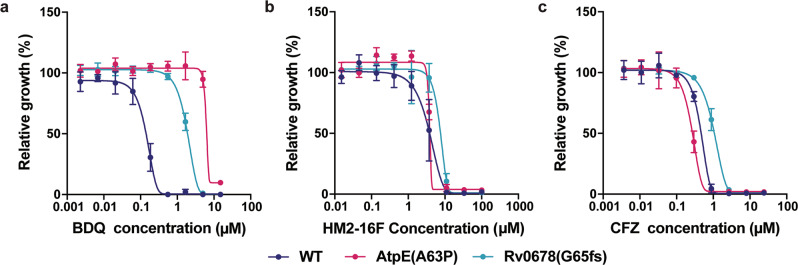
Bedaquiline-resistant mutants of *M. tuberculosis* are not cross-resistant to HM2-16F. The *M. tuberculosis* strains mc^2^6206 (WT), AtpE(A63P), and Rv0678(G65fs) were grown in the presence of increasing concentrations of: **a** BDQ, **b** HM2-16F or **c** clofazimine and growth measured after 10 days. Error bars represent standard deviation from four biological independent experiments.

The structure of the *M. phlei c*-ring bound to BDQ revealed that BDQ mimics the *a*-subunit arginine, which temporarily interacts with the *c*-ring Glu65 carboxylate side chain during the ion translocation cycle^8^. Although BDQ and HM2-16F both have arginine mimetic groups, HM2-16F is otherwise structurally dissimilar. We, therefore, sought to understand the mechanism of HM2-16F and its dissimilarity to BDQ. Firstly, we attempted to isolate HM2-16F-resistant mutants in *M. tuberculosis* mc^2^6230. While mutants were able to be isolated at a frequency of 1.45 × 10^−6^, these did not show mutations in the F_1_F_o_-ATP synthase. Instead, we identified 4 separate mutations in the transcription factor Rv3066 (R38S, G134fs, G157R and E173*), which regulates the Mmr efflux pump, with these mutants showing ~4-fold increases in MBC to HM2-16F (Supplementary Figure 4). Notably, Mmr is not reported to contribute to efflux-mediated BDQ resistance, which is typically ascribed to the MmpL5 pump (regulated by Rv0678)^29^. Furthermore, HM2-16F cross-resistance was not observed in a BDQ-resistant mutant carrying a mutation in the ATP synthase *c*-ring subunit (AtpE(A63P); Fig. 4 and Table 2) or in the efflux pump regulator Rv0678 (Rv0678(G65fs)), which is cross-resistant to clofazimine^29^. Taken together, these results suggests that HM2-16F has only a weak interaction with the F_1_F_o_-ATP synthase that may not necessarily drive its primary mode of action. To understand the basis of this difference, we performed molecular docking simulations with HM2-16F against the recently solved *M. phlei* F_1_F_o_-ATP synthase^8^. The acidic glutamate residue (E65; *M. phlei* numbering) was used as the centre of the binding site as well as a hydrogen-bonding constraint on the assumption that the acylguanidine of HM2-16F mimics the binding of the basic dimethylamine in BDQ. The docked structure (Fig. 3e and Supplementary Fig. 5) suggests that HM2-16F binds to the F_1_F_o_-ATP synthase in a very different way to BDQ (e.g., additional interactions with F58; *M. phlei* numbering) that may reduce its affinity for this binding site. The putative binding pose suggests that mutations affecting BDQ binding might be tolerated by HM2-16F, consistent with our mutational data (Fig. 4).

**Table 2:** Minimum inhibitory concentrations (MIC, μM) of selected inhibitors against wild-type and mutant *M. tuberculosis* mc^2^6206 strains.

Compound	WT	AtpE(A63P)	Rv0678(G65fs)
BDQ	0.3	8	4
HM2-16F	9	5	12
Clofazimine	0.9	0.5	3

All MIC values are reported in micromolar final concentration. All strains are listed in Supplementary Table 1. WT = wild-type *M. tuberculosis* mc^2^6206.

### Metabolite profiles of *M. tuberculosis* treated with HM2-16F

To identify alternative targets of HM2-16F, we challenged *M. tuberculosis* H37Rv with varying concentrations of HM2-16F (1–10× MIC) and identified changes in intracellular metabolites by LC/MS-MS (Supplementary Fig. 6). We considered two different scenarios for biological significance: (1) sub-saturated changes in metabolites, where changes are in a dose-dependent manner^30^ and (2) saturated changes, where the three measurements showed a consistent fold-change in metabolites regardless of HM2-16F concentration.

**Fig. 5:**
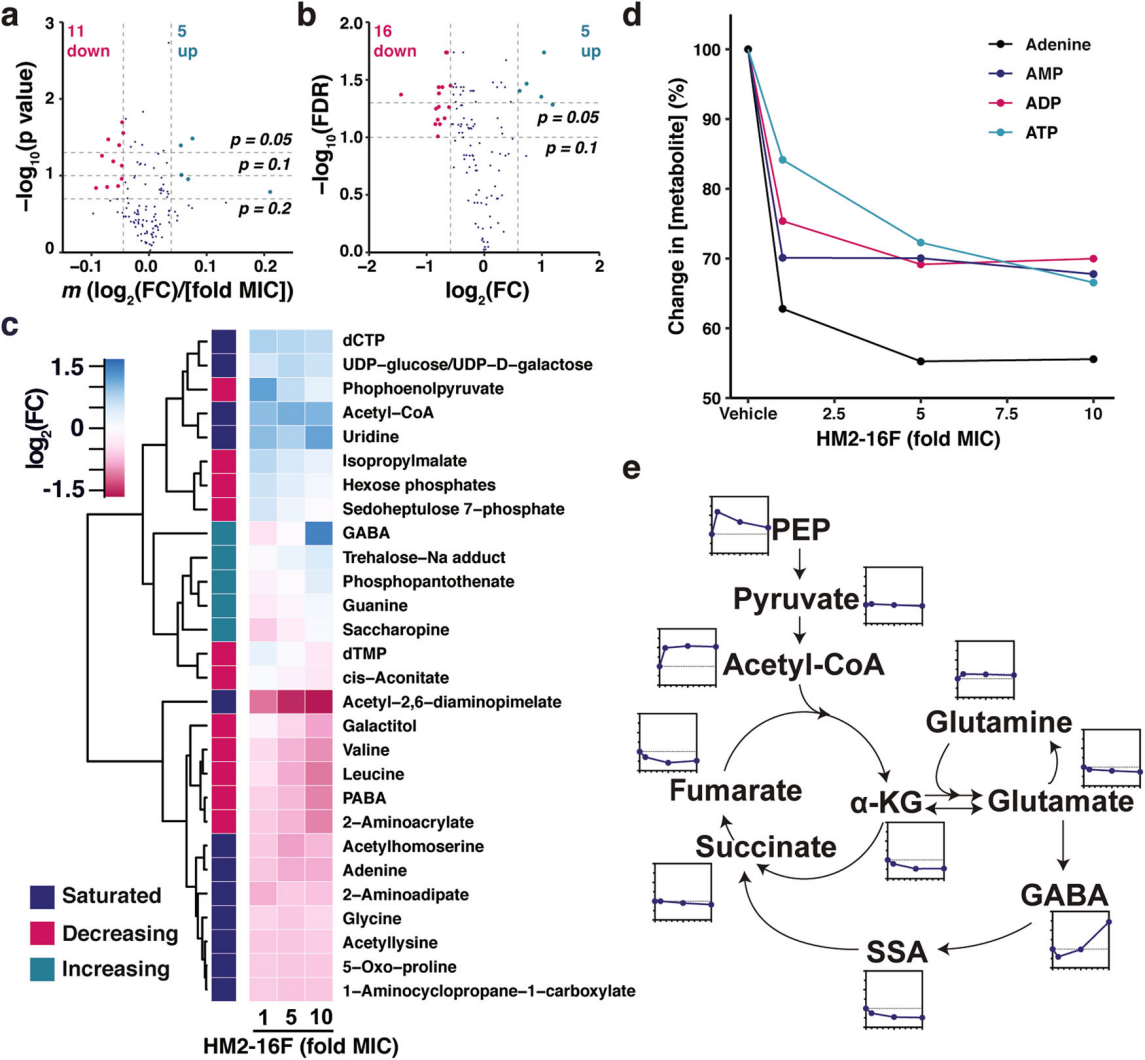
Metabolomic profiling of HM2-16F-treated cultures of *M. tuberculosis* indicates HM2-16F inhibits respiration and induces cellular reductive stress. **a** Global profile of metabolites with dose-dependent changes, as assessed by log-linear regressions. **b** Global profile of metabolites with dose-independent changes, as assessed by two sample t-tests with Benjamini & Hochberg False Discovery Rate (FDR) adjustment. **c** Fold changes of selected metabolites, classified according to whether the metabolite changed in a dose-dependent (increasing/decreasing) or dose-independent manner. **d** Changes in adenine nucleotide/nucleoside pools. **e** Relative changes in the indicated metabolites. Raw data are available in Supplementary Data 1.

HM2-16F caused a saturating or sub-saturating change in 37 metabolites (Fig. 5a, b and Supplementary Data 1). Of the sub-saturated effects, 11 metabolites decreased in a dose-dependent manner, while 5 were increased (Fig. 5a, c and Supplementary Data 1). For saturated effects, HM2-16F caused a consistent decrease in 16 metabolites, while 5 metabolites increased (Fig. 5b, c and Supplementary Data 1). HM2-16F affected the total nucleotide pool in *M. tuberculosis:* ATP, ADP, AMP, and adenine concentrations were all decreased (Fig. 5c, d), while CTP and uridine showed a saturated increase (Fig. 5c). Guanine concentrations increased in a dose-dependent manner, while TMP decreased (Fig. 5c). These changes suggest an inability to regenerate ATP and impaired biosynthesis of nucleoside pools.

Additionally, HM2-16F caused a reduction of carbon entry into the TCA cycle. Acetyl-CoA was consistently increased at each concentration of HM2-16F (Fig. 5c, e), phosphoenolpyruvate (PEP) was initially increased, but the response declined with increasing concentrations of HM2-16F (Fig. 5c, e). Collectively, these data indicate that flux through the oxidative decarboxylation steps of the TCA cycle was reduced. In support of this, γ-aminobutyric acid (GABA) increased in a dose-dependent manner (Fig. 5c, e), suggesting that there was lower flux in the GABA shunt, a major pathway for bypassing steps in the TCA cycle in *M. tuberculosis*^31^. Together with the depletion of ATP:ADP, this suggests that F_1_F_o_-ATP synthase inhibition, mediated by HM2-16F, has resulted in either consequent or concomitant inhibition of the electron transport chain and cellular reductive stress that feedback onto energy cofactors, reducing equivalents and alternative central metabolic pathways.

### Cytochrome *bd* inhibition enables HM2-16F to sterilize *M. tuberculosis* cultures in combination with Q203

**Fig. 6:**
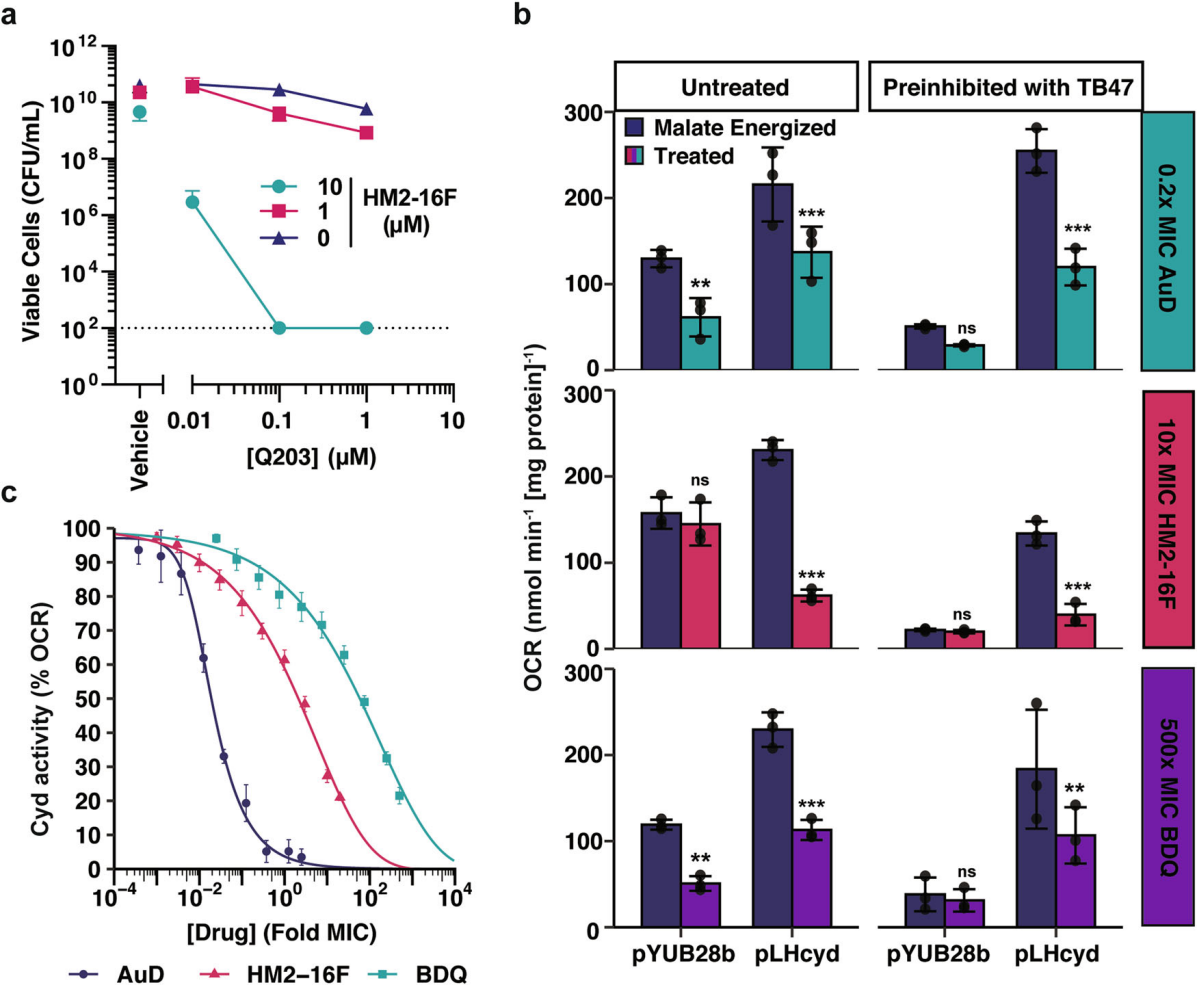
HM2-16F synergizes with Q203 by inhibiting cytochrome *bd* oxidase. **a** Cell killing (viability) of *M. tuberculosis* with increasing amounts of both Q203 and HM2-16F after 14 days challenge with both compounds. Error bars indicate standard deviation from three independent experiments. **b** Inhibition of the *M. tuberculosis* cytochrome *bd* oxidase in IMVs from *M. smegmatis* mc^2^155 Δ*cydAB* with either pYUB28b (empty vector) or pLHcyd- *Mtb*CydABDC^+^. IMVs were pre-inhibited with 500 nM TB47 for 1 min as indicated. Oxygen consumption was initiated with 5 mM malate (final concentration) as the sole electron donor. The indicated treatment: 0.2× MIC aurachin D (AuD) (top panel 6b), 10× MIC HM2-16F (middle panel **b**) and 500× MIC BDQ (bottom panel **b**) was added once malate energized oxygen consumption reached steady state (~2 min after malate addition). Error bars represent standard deviation from triplicate measurements. Asterisks indicate significance compared to the corresponding malate energized OCR *** = *p* < 0.001, ** = *p* < 0.01, ns = *p* > 0.05 (One-way ANOVA, Tukey multiple comparisons, 95% CI). **c** Titration of the indicated compounds in malate-energized IMVs of *M. smegmatis* mc^2^155 Δ*cydAB* pLHcyd- *Mtb*CydABDC^+^ pre-inhibited with 500 nM TB47 (final concentration). Error bars indicate standard error from four technical replicates.

As the metabolomic profiling suggested induction of reductive stress, we next examined the potential of HM2-16F to synergize with inhibitors of other protein complexes in the *M. tuberculosis* respiratory pathway. Clear evidence of HM2-16F synergy was observed with the quinol:cytochrome *c* oxidoreductase inhibitor Q203 (Fig. 6). Treatment of *M. tuberculosis* mc^2^6230 with 2.5-fold MIC of HM2-16F effectively sterilized cultures in the presence of 10-fold MIC of Q203 (Fig. 6a, limit of detection of 100 CFU mL^−1^). The combination is more potent than Q203 alone (Fig. 6a). In contrast, BDQ is not synergistic with Q203^23^, suggesting that HM2-16F interacts with other cellular targets that synergize with inhibition of the cytochrome *bcc*-*aa*_3_ oxidase.

Recently, we demonstrated that the cytochrome *bd* inhibitor ND-011992 potently synergizes with Q203 to induce bactericidal killing^22^. We, therefore, hypothesized that HM2-16F might also inhibit cytochrome *bd* oxidase. We assessed the ability of HM2-16F to inhibit *M. tuberculosis* cytochrome *bd* (CydABDC^+^) heterologously expressed in a markerless *M. smegmatis* cytochrome *bd* mutant (Δ*cyd* pLHcyd-*Mtb*CydABDC^+^) (Supplementary Table 1)^32^. IMVs prepared from *M. smegmatis* ∆*cydAB* harbouring an empty vector control (pYUB28b) exhibited high rates of oxygen consumption when energized with malate (Fig. 6b). When *M. smegmatis* ∆*cydAB* was complemented with the *cydABDC* operon from *M. tuberculosis* (*Mtb*CydABDC^+^) the OCR increased significantly suggesting the cytochrome *bd* oxidase from *M. tuberculosis* was functional in *M. smegmatis* (Fig. 6b). When HM2-16F (10× MIC) was added to malate-energized IMVs of *M. smegmatis* ∆*cydAB-*pYUB28b no significant effect was observed on the OCR (Fig. 6b, top panel). However, addition of HM2-16F to *M. smegmatis* ∆*cydAB-Mtb*CydABDC^+^ caused significant inhibition of the OCR suggesting this inhibition was dependent on the presence of cytochrome *bd*. When IMVs were preincubated with the potent cytochrome *bcc*-*aa*_3_ oxidase inhibitor TB47^30^, the OCR in the *M. smegmatis* ∆*cydAB-*pYUB28b mutant was completely inhibited demonstrating that oxygen consumption in this genetic background was entirely mediated by cytochrome *bcc*-*aa*_3_ oxidase. Oxygen consumption was restored in *M. smegmatis* ∆*cydAB-Mtb*CydABDC^+^ that was TB47-insensitive as this was mediated by cytochrome bd, and HM2-16F addition inhibited this OCR (Fig. 6b, top panel). The combination of TB47 with HM2-16F was effective in shutting down the OCR of *M. smegmatis* ∆*cydAB-Mtb*CydABDC^+^. The same experiments were performed with the specific ATP synthase inhibitor bedaquiline (Fig. 6b, middle panel) and the known cytochrome *bd* oxidase inhibitor, aurachin D^32^ (Fig. 6b, bottom panel). In contrast to HM2-16F that exhibited a remarkable specificity for cytochrome *bd*, bedaquiline and aurachin D showed inhibition of OCR in the *M. smegmatis* ∆*cydAB-*pYUB28b genetic background. This inhibition of OCR was observed throughout the other treatments suggesting both inhibitors impacted negatively on the OCR in a cytochrome *bd*-independent manner (Fig. 6b, middle and bottom panel).

HM2-16F was found to inhibit cytochrome *bd* activity with an IC_50_ of 21.2 μM (Supplementary Table 2; Fig. 6c), close to its MIC and comparable to the concentrations used in synergy experiments (Fig. 6A). BDQ was also able to inhibit cytochrome *bd* (Fig. 6c and Supplementary Table 2), however, the concentrations required were orders of magnitude higher than its MIC and not as specific towards cytochrome *bd* as HM2-16F (Fig. 6b). The effects of BDQ are likely not relevant to its bacteriostatic or bactericidal action. These findings support that HM2-16F synergizes with Q203 through direct cytochrome *bd* inhibition.

## Discussion

The F_1_F_o_-ATP synthase of *M. tuberculosis* is a validated drug target, as demonstrated by the clinical approval of BDQ and of its utility in treating drug-resistant tuberculosis infection. Nevertheless, concerns about BDQ’s safety^6^ and the isolation of resistant mutants^12,13^ highlight the need for next-generation inhibitors. Amilorides are a class originally identified in 1967 as potassium-sparing diuretics that act on ENaCs in the kidney^26^. Numerous studies since then have shown that amilorides have a multitude of other activities resulting from their arginine-mimetic acylguanidine moiety^27,33–36^. We postulated that the acylguanidine of amilorides could make them F_1_F_o_-ATP synthase inhibitors since it is known that the dimethylamino group of BDQ functions as an arginine mimetic in its interaction with F_1_F_o_-ATP synthase c-ring^7^. In this study, we found that the 6-substituted amiloride derivative HM2-16F functions weakly as an *M. tuberculosis* F_1_F_o_-ATP synthase inhibitor, but more potently functions as an inhibitor of the *M. tuberculosis* cytochrome *bd* oxidase. With no diuretic activity and better *M. tuberculosis* inhibitory activity than amiloride, we propose that 6-substituted amiloride derivatives like HM2-16F present a promising scaffold for developing alternative drugs to BDQ with different resistance profiles.

Arginine mimetics are present in a broad range of compounds. Not all arginine mimics could be F_1_F_o_-ATP synthase inhibitors as this would be inconsistent with the favourable selectivity indices that have been obtained for BDQ^7^, which itself has a lower structural resemblance to the arginine side chain than amilorides or other aryl/alkylguanidines. Instead, and in line with the BDQ-bound structure of the *M. phlei* c-ring^8^, we propose that steric hindrance at the membrane-embedded a–c subunit interface selects for only certain guanidine-mimetic chemotypes. Along these lines, amiloride derivatives targeting the *M. tuberculosis* F_1_F_o_-ATP synthase may possess a similar range of selectivity. In support of this, AtpE^A63P^ mutants were not cross-resistant to HM2-16F and this likely indicates that HM2-16F does not utilize the Asp^32^ water-mediated bonding network to efficiently bind to the c-ring, unlike BDQ^8^.

The effect of HM2-16F on total cellular metabolites showed many parallels to previous metabolomic and transcriptomic profiling of BDQ^37^. For example, GABA was found to increase in abundance following BDQ treatment on a timescale comparable to our metabolomic profiling^37^. Additionally, one of the *M. smegmatis* pyruvate dehydrogenases (MSMEG_4712) is upregulated 12-fold in response to BDQ^17^, consistent with our observation of increased abundance of acetyl-CoA following HM2-16F treatment. Finally, proteomic analysis of BDQ-treated *M. tuberculosis* found a time-dependent increase in abundance of PEP carboxykinase (Rv0211^15^), consistent with the increased abundance of PEP seen with HM2-16F. Taken together, these data suggest that the effects of HM2-16F and BDQ are comparable at a metabolic level. In conjunction with our CRISPRi interference data of the *atp* operon knockdown, this may suggest that HM2-16F has an ability to indirectly affect the F_1_F_o_-ATP synthase or directly targets a different molecular site of the F_1_F_o_-ATP synthase. More studies are required to understand the direct or indirect interaction of HM2-16F with the mycobacterial F_1_F_o_-ATP synthase.

**Fig. 7:**
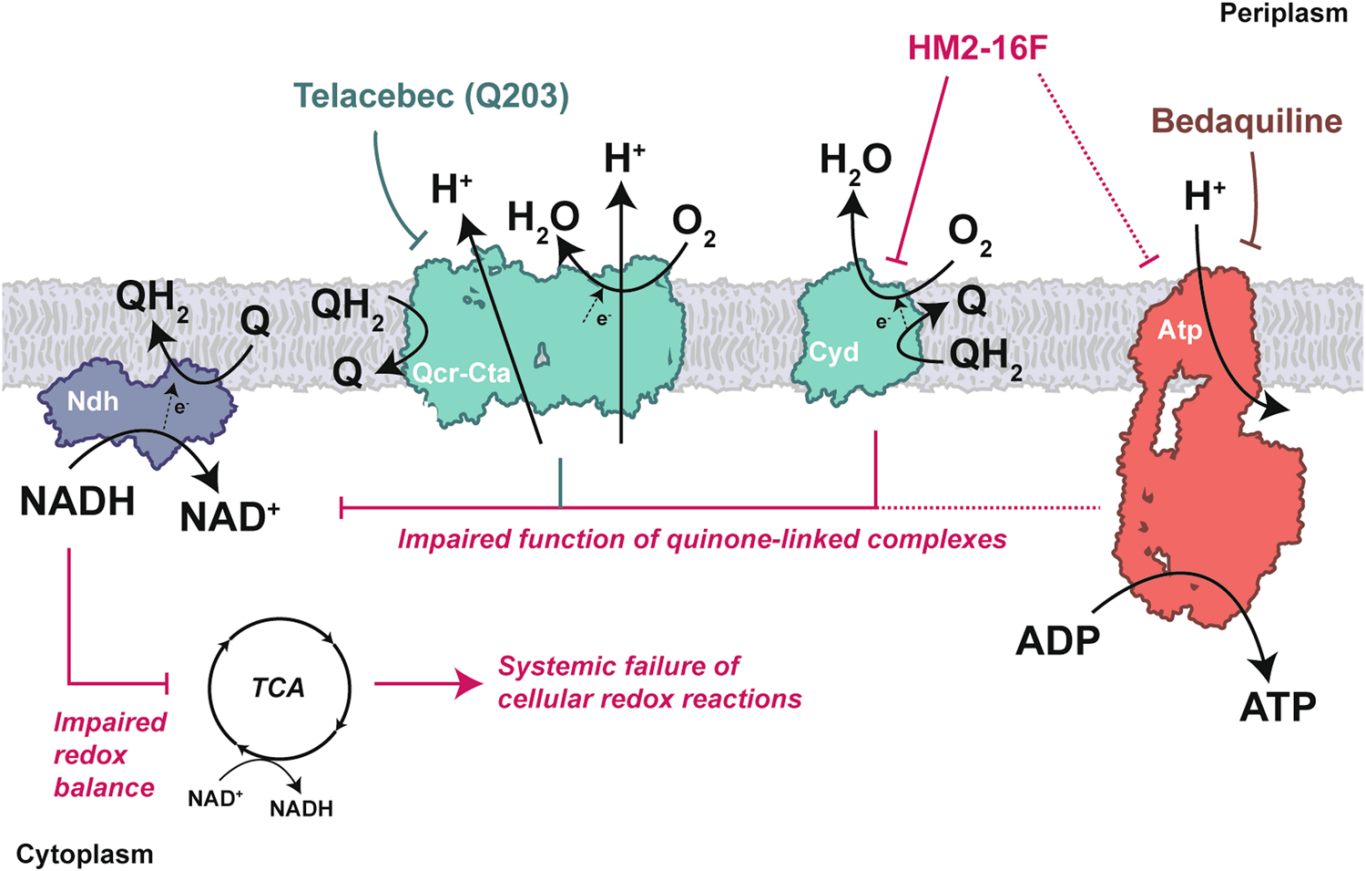
Summary of the action of HM2-16F at the level of the respiratory chain. HM2-16F inhibits cytochrome *bd* and, to a lesser or indirect extent, the F_1_F_o_-ATP synthase. Disruption of quinol regeneration can affect the primary dehydrogenases of the respiratory chain leading to dysregulated regeneration of reducing equivalents that can lead to a systemic failure of cellular redox reactions. The addition of Q203 (or TB47), direct inhibitors of the cytochrome *bcc-aa*_3_ oxidase branch, removes the only alternative pathway that mycobacteria can use to escape this inhibition of oxygen consumption leading to cell death.

The potent synergy seen with HM2-16F and Q203 serves as a major point of difference to BDQ. BDQ can function as an ionophore^17,18,38^ and is not synergistic with Q203 in vitro^23^. However, HM2-16F displayed strong inhibition of the cytochrome *bd* oxidase that allowed it to affect synergistic killing with TB47, a validated inhibitor of the cytochrome *bcc*-*aa*_3_ oxidase^30^. Overall, we propose a model (Fig. 7) where HM2-16F inhibits cytochrome *bd* and, to a lesser or indirect extent, the F_1_F_o_-ATP synthase. Disruption of quinol regeneration can affect the primary dehydrogenases of the respiratory chain, leading to dysregulated regeneration of reducing equivalents that can lead to a systemic failure of cellular redox reactions. The addition of TB47 (or Q203 – Telacebec) removes the only alternative pathway that mycobacteria can use to escape this inhibition of oxygen consumption leading to cell death (Fig. 7). HM2-16F could create alternative treatment options for patients unable to take BDQ, such as those taking other drugs that prolong the QT interval^39^. Overall, we propose that amilorides are a promising class for developing an alternative to BDQ where toxicity or resistance profiles might preclude BDQ’s usage. In this regard, the amiloride scaffold appeals as an ideal starting point for selective optimization of side activity programs aimed at producing new respiratory inhibitors and antimycobacterial drugs.

## Methods

### Bacterial strains, media, and growth conditions

Bacterial strains used in this study are listed in Supplementary Table 1. For *M. smegmatis* mc^2^155 growth, cells were grown in modified Hartman’s de Bont medium^40^, containing 0.2% glycerol as the sole carbon and energy source. Tween80 was omitted for determination of the MIC. For *M. tuberculosis* strains mc^2^6230^41^ and mc^2^6206^42^ growth (Supplementary Table 1), cells were grown in Middlebrook 7H9 broth supplemented with OADC (0.005% oleic acid, 0.5% bovine serum albumin, 0.2% dextrose, 0.085% catalase), 0.05% tyloxapol (Sigma) and 25 μg/mL pantothenic acid. The growth of mc^2^6206 was also supplemented with leucine (50 µg/mL). Unless otherwise specified, the following growth procedures were followed: *M. smegmatis* was grown in 125 mL conical flasks, containing 25 mL media, and *M. tuberculosis* was grown in 30 mL inkwells, containing 10 mL media. *M. smegmatis* and *M. tuberculosis* were grown with agitation at 200 rpm and 140 rpm respectively. All cultures were maintained at 37 °C. To obtain hypoxic cultures of *M. tuberculosis*, 30 mL of complete 7H9 media was inoculated at an optical density (OD_600_) of 0.05 in 100 mL serum vials (Wheaton) that were stopped and capped^40^. Cultures were monitored until methylene blue (1.5 μg/mL) control indicator decolorized to signify cells has reached hypoxia (~7–10 Days). Once cultures became hypoxic, cells were challenged with BDQ and HM2-16F at 10× MIC and 5× MIC respectively with the inclusion of a DMSO only control, all experiment were done in biological triplicate. Colony counts (cfu/ml) to determine cell viability were taken on days 0, 3, 7, 14, 21, and 28 after compound challenge and plated on complete 7H11 agar.

### Inhibition and killing assays

MIC values were performed by serial dilution in 96-well plates in growth medium. Unless otherwise specified, MIC is defined as the lowest concentration to inhibit all observable growth. Log phase cultures were used to inoculate each dilution to a final OD600 of 0.005. Cultures were incubated at 37 °C, 200 rpm. Unless otherwise specified, the MIC was determined from the visual presence or absence of growth after 2 days (*M. smegmatis*), 7 days (*M. tuberculosis* H37Rv), 10 days (*M. tuberculosis* mc^2^6206) or 1 day (all other strains) (Supplementary Table 1). Controls containing no compound or compound dissolution solvent (DMSO) alone were included in all experiments.

### Infection of differentiated human THP-1 macrophages and drug susceptibility assays ex vivo

The human monocytic cell line THP-1 was cultured in standard RPMI 1640 macrophage medium supplemented with 10% inactivated fetal bovine serum and 1 mM sodium pyruvate at 37 °C with 5% CO_2_^43^. THP-1 monocytes (5 × 10^5^ cells/well) were differentiated overnight using 20 ng/mL phorbol myristate acetate (PMA) and seeded in a 96 well-plate. The next day, differentiated macrophages were infected with a mid-logarithmic phase culture of *Mycobacterium bovis* BCG (Pasteur 1173P2)^44^ (OD 0.4–0.8) at a multiplicity of infection of 10:1 (10 bacteria/1 cell). Infection was allowed to proceed for 5 h. Cells were then washed 4 times with pre-warmed complete RPMI to remove extracellular bacilli. RPMI media containing compounds at varying concentrations was added to the infected cells and incubated for 1–3 days at 37 °C with 5% CO_2_. At various times, the infected cells were lysed in distilled water containing 1% Tween80 for 5 min at room temperature to determine the number of CFU/mL on Middlebrook 7H11 + OADC agar. All cells used as inocula were washed in saline (0.85% NaCl). To rule out adverse effects on THP-1 cell physiology leading to indirect effects on *M. bovis* BCG replication in these cells, Alamar Blue assays^45^ were performed to determine if HM2-16F was toxic to THP-1 cells. THP-1 cells were grown in 96-well plates and treated with compounds at a range of concentrations (0.05–500 μM) for 48 h. The percentage of cell viability was determined by subtracting values for non-treated infected cells from the non-infected cells.

### Isolation of bedaquiline and HM2-16F resistant mutants

*M. tuberculosis* mc^2^6230 resistant mutants against HM2-16F and mc^2^6206 resistant mutants against BDQ were isolated as follows^46^. Cultures were grown for approximately 2 weeks, in Middlebrook 7H9 + OADC medium, to an OD_600_ of approx. 0.4–1 (i.e., ~1 × 10^8^ CFU/mL). Cells (100 µL) at approximate densities of 1 × 10^8^, 1 × 10^7^, 1 × 10^6^ and 1 × 10^5^ CFU/mL were plated on 7H11 + OADC agar containing either 20 µM HM2-16F (5x the solid media MIC) or 1, 3, or 11 µM BDQ (3, 9, and 30× MIC). Plates were incubated at 37 °C until colonies appeared, approximately 4 weeks.

### Whole Genome sequencing and single nucleotide polymorphism identification

Genomic DNA was extracted from *M. tuberculosis* using the MoBio UltraClean Microbial DNA isolation Kit. Ten mL of late log phase culture was harvested, resuspended in 300 µL MoBio bead binding buffer, and heat-inactivated for 15 min at 95 °C. Manufacturer’s guidelines were followed to extract genomic DNA from the heat-inactivated culture. Whole-genome sequencing (Illumina 150 bp paired-end reads) was performed at Otago Genomics and reads were mapped to the wild type H37RV genome using Geneious.

### Antimicrobial susceptibility of *atpB* transcriptional knockdown strains

Sensitivity of *M. tuberculosis* (mc^2^6230) strains expressing an sgRNA targeting *atpB*^28^ or non-targeting control^47^ to HM2-16F were determined. Briefly, cultures were inoculated into 96-well plates containing supplemented Middlebrook 7H9 broth (OADC + PAN + KAN), at a starting OD_600_ of 0.005, in a total starting volume of 100 µL. Anhydrotetracycline (ATc) at 0, 10, or 100 ng/mL was added to alternating rows, and HM2-16F was dispensed from a 9-point, three-fold dilution gradient to each well, with a maximum of 2% DMSO present. Ninety-six-well plates were incubated without shaking at 37 °C for 10 days. On day 10, OD_600_ values were measured in a Varioskan LUX microplate reader, and minimum inhibitory concentration (MIC) values were determined. To determine the effects on bacterial viability, culture was removed from desired wells on day 10 and diluted along a four-point, ten-fold dilution curve. Five µL was spotted onto 7H11 media, incubated at 37 °C and colony-forming units were determined after four weeks.

### MALDI-MS-based competition studies of ATP synthase inhibitors with *N*,*N*^’^-dicyclohexylcarbodiimide (DCCD)

Purified *M. phlei* c-ring in 0.6% lauryldimethylamine oxide (LDAO) (Sigma)^8^ was used to assay the competition of dicyclohexylcarbodiimide (DCCD) with BDQ or HM2-16F. The concentrated sample (6.5 mg/mL) was diluted to 0.1 mg/mL using 20 mM cacodylate/trimethylamine/NH_3_ (pH 7.5). Compounds, solubilized in DMSO, were added to the indicated final concentrations. Samples were incubated for 1 h at room temperature before adding 25 mM DCCD. Aliquots were removed at several time points (0–45 min), directly mixed in a 1:1 ratio with 2′,4′,-dihydroxyacetophenone matrix, and applied onto a ground steel MALDI target in duplicates. MALDI mass spectra were acquired in the mass range of 5–20 kDa on a Bruker Autoflex III Smartbeam MALDI-TOF mass spectrometer using optimized ionization, ion optics, and detector settings. Spectra were recalibrated using the near-neighbour method with a calibrant mixture (Bruker Protein Calibration standard 1, Bruker Daltonics, Bremen). All spectra were evaluated and recalibrated using the software Bruker FlexAnalysis 3.3 (Build 75). After background substraction (TopHat), smoothing (Savitzky-Golay, width 1 m/z, 3 cycles) and peak picking (Centroid, s/n 5) the intensities of the c-monomer and the DCCD-labelled species were used for calculating the efficiency of DCCD binding. The “labelling efficiency” was calculated as the intensity ratio of the DCCD-bound species to the sum of the labelled and unlabelled species. All values were calculated from 3 to 4 experimental replications and technical MALDI measurement duplicates.

### Molecular docking

The 3D structure of HM2-16F was created and minimized using Avogadro (v1.2.0)^48^. Docking was carried out using GOLD v5.7.2. Compounds were docked into the BDQ binding site of the ATP synthase c-ring structure (PDB ID: 4V1F,^8^) with Glu65 used as the centre of the binding site as well as a protein hydrogen bonding constraint. Nine poses were produced and 3 fell within 1.5 Å RMSD. Poses that failed to interact with E65 were excluded.

### Liquid chromatography-mass spectrometry (LC-MS) metabolomics

Filter-cultured *M. tuberculosis* strain H37Rv was first grown for 5 days in 7H10 agar media to expand biomass, and then moved to fresh 7H10 medium containing compounds or a vehicle control (DMSO) for a 24 h exposure^30^. *M. tuberculosis* metabolism was quenched by plunging *M. tuberculosis*–laden filters into extraction buffer (acetonitrile: methanol: H_2_O = 40:40:20), which was precooled to −40 °C on dry ice^49^. *M. tuberculosis* metabolites were then extracted by mechanical lysis with zirconia beads in Precellys tissue homogenizer under continuous cooling at or below 2 °C. Extracted *M. tuberculosis* metabolites were analyzed by high performance liquid chromatography-coupled mass spectrometry using an Agilent 1290 HPLC and Accurate Mass 6220 TOF or 6520 qTOF mass spectrometer^50^. *M. tuberculosis* metabolites were identified based on curated accurate mass-retention time identifiers, and quantified using Agilent Quantitative Analysis software and Agilent Profinder software with a mass tolerance of < 0.005 Da.

### Oxygen consumption in inverted membranes vesicles (IMVs)

IMVs of the *M. smegmatis* mc^2^155 Δ*cyd* pLHcyd (overproducing cytochrome *bd* oxidase from *M. tuberculosis)*, or the corresponding empty vector control (Δ*cyd* pYUB28b) (Supplementary Table 1) were prepared as follows. A single colony of the production strain (Δ*cyd* pLHcyd or Δ*cyd* pYUB28b) was used to inoculate LBT media containing 50 µg/ml hygromycin B. This culture was grown for 72 h at 37 °C and 200 rpm to achieve maximum cytochrome *bd* oxidase production and cells were harvested by centrifugation at 4000 × *g* for 20 min at 4 °C. Cells were resuspended in buffer containing 50 mM Tris-HCl (pH 7.4), 5 mM magnesium chloride, 0.05% Tween 80 and 0.1 mg/ml Pefabloc SC (5 ml buffer/1 g cells). Cells were disrupted using a high-pressure homogenizer (Avestin Emulsiflex C3) via several passages (4–6) at 22,000 psi. The lysate was centrifuged at 10,000 × *g* for 20 min to remove cell debris and unlysed cells. The supernatant of the low-speed centrifugation step was subsequently centrifuged at 225,000 × *g* for 90 min. Pelleted IMVs were resuspended in buffer (50 mM Tris, 100 mM KCl, 5 mM MgCl_2_, pH 7.5) and 5 mM malate was used to initiate oxygen consumption that was measured using an Oroboros O2k fluorespirometer. For titrations, 500 nM TB47 (an inhibitor of cytochrome *bcc*:*aa*_3_ oxidase complex^30^) was added prior to the experiment. IMVs were used at a protein concentration 12.5  μg mL^−1^. Stepwise titrations were performed using the Oroboros O2k TIP2k automatic injection micropump, with rates measured at 120 s intervals between each injection. Data are normalized to protein concentrations that were estimated by BCA assay (Thermo), using a BSA standard.

### ATP synthesis in inverted membrane vesicles

ATP synthesis in IMVs was carried out at 37 °C in 1 ml of 50 mM Tris-HCl (pH 8.0) buffer containing 5 mM MgCl_2_ and 100 mM KCl with constant stirring. Approximately 0.1 mg mL^−1^ of IMVs were incubated with stirring at 37 °C for 2 min, followed by incubation in the presence of 1 mM NADH for 2 min. When performing inhibition experiments, test inhibitor was added 5 min prior to the addition of NADH. ATP synthesis was initiated with the concurrent addition of 0.75 mM ADP and 2.5 mM potassium phosphate (pH 8.0). At various time intervals, 100 µl aliquots were removed and transferred to 400 µl of stop solution (1% trichloroacetic acid, 2 mM EDTA plus 200 µM CCCP). Each sample was diluted 500-fold in water prior to the measurement of ATP. The amount of ATP was determined by the luciferin-luciferase assay as follows. Each sample was diluted into 400 µl of Tris acetate buffer (50 mM Tris acetate, pH 7.8, 2 mM EDTA, 50 mM MgCl_2_) in a luminometer tube. Luciferin-luciferase reagent (50 µL, Sigma) was added to the tube, and the fluorescence monitored with a chemiluminometer (FB 12 luminometer; Berthold)^51^. The amount of ATP synthesized was calculated from a standard curve performed on the day of each set of ATP measurements. For each individual experimental set, the presence of background ATP was measured using non-energized vesicles (no NADHJ) and subtracted from total ATP measured.

### Statistics and reproducibility

All statistical analysis was performed in R version 4.0.2. IC_50_ values and 95% confidence intervals were determined by fitting 4-parameter logistic regressions using the package *nplr*. Pairwise comparison and multiple comparison corrections were performed using estimated marginal means in the package *emmeans*. For metabolomics analysis, linear regressions were modelled to the log_2_–log_10_ transformed data (i.e., log_2_(Fold Change) vs log_10_([Drug])) for each metabolite. The *p*-value derived from the F-statistic was used to evaluate if the two variables were linearly related. Otherwise, t-test comparisons with Benjamini & Hochberg False Discovery Rate adjustments were performed.

### Reporting summary

Further information on research design is available in the Nature Research Reporting Summary linked to this article.

## Supplementary information

Supplementary Information (new)

Description of additional Supplementary Files (new)

Supplementary Data 1

Supplementary Data 2

Reporting Summary

## Data Availability

Raw metabolomic data are presented in Supplementary Data 1. Source data underlying main figures are presented in Supplementary Data 2. Sequencing data are available from NCBI BioProject under the accession numbers PRJNA802611 and PRJNA802880. All other data are available from the corresponding author upon reasonable request.
