# Supplementary material for: An amiloride derivative is active against the F1Fo-ATP synthase and cytochrome bd oxidase of Mycobacterium tuberculosis
Source: Commun Biol. 2022 Feb 24;5:166. doi: 10.1038/s42003-022-03110-8 (PMC8873251; doi:10.1038/s42003-022-03110-8)
Supplement: Supplementary file 1 — Supplementary Information (new) [file 42003_2022_3110_MOESM1_ESM.pdf]

Supplementary Information for:

An amiloride derivative is active against the F_1F_o-ATP synthase and cytochrome *bd* oxidase of *Mycobacterium tuberculosis*

Kiel Hards[1,2*], Chen-Yi Cheung[1*], Natalie Waller[1], Cara Adolph[1], Laura Keighley[1], Zhi Shean Tee[1], Liam K. Harold[1,2], Ayana Menorca[1], Richard S. Bujaroski[3,4], Benjamin J. Buckley[3,4], Joel D. A. Tyndall[5], Matthew B. McNeil[1,2], Kyu Y. Rhee[6], Helen K. Opel-Reading[7], Kurt Krause[2,7], Laura Preiss[8,9], Julian D. Langer[10], Thomas Meier[11,12], Erik J. Hasenoehrl[13], Michael Berney[13], Michael J. Kelso[3,4] and Gregory M. Cook[1,2]

1. Department of Microbiology and Immunology, University of Otago, Dunedin, New Zealand
2. Maurice Wilkins Centre for Molecular Biodiscovery, The University of Otago, Dunedin, New Zealand
3. Molecular Horizons and School of Chemistry and Molecular Bioscience, University of Wollongong, Wollongong, Australia
4. Illawarra Health and Medical Research Institute, Wollongong, Australia
5. School of Pharmacy, University of Otago, Dunedin, New Zealand
6. Weill Department of Medicine, Weill Cornell Medical College, New York, United States
7. Department of Biochemistry, University of Otago, Dunedin, New Zealand
8. Department of Structural Biology, Max-Planck Institute of Biophysics, Frankfurt am Main, Germany
9. Octapharma Biopharmaceuticals GmbH, Heidelberg, Germany
10. Department of Molecular Membrane Biology, Max-Planck Institute of Biophysics, Frankfurt am Main, Germany
11. Department of Life Sciences, Imperial College London, London, United Kingdom
12. Private University in the Principality of Liechtenstein, Triesen, Liechtenstein
13. Department of Microbiology and Immunology, Albert Einstein College of Medicine, New York, USA

*These authors contributed equally

Address correspondence to: Greg.cook@otago.ac.nz or mkelso@uow.edu.au

Keywords: Tuberculosis, F_1F_o-ATP synthase, cytochrome *bd*, antibiotics

Supplementary Table 1: Bacterial strains and plasmids used in this study

Strain or Plasmid	Description	Source
E. coli strains		
ATCC 10536	*E. coli* quality control strain	ATCC
S. aureus strains		
ATCC 6538	*S. aureus* quality control strain	ATCC
E. faecalis strains		
JH2-2	Laboratory strain, plasmid-free; rif^r , fs^r	[1]
M. smegmatis strains		
mc^{2}155	Electrocompetent wild-type strain of *M. smegmatis*	[2]
Δ*cyd*	mc^{2}155 with a markerless in frame deletion in the *cydAB* gene	[3]
Δ*qcr*	mc^{2}155 with a markerless in frame deletion in the *qcrB* gene	[4]
M. tuberculosis strains		
H37Rv	*M. tuberculosis* reference strain	
mc^{2}6230	Avirulent auxotrophic *M. tuberculosis* mutant (Δ*RD1* Δ*panCD*). Wild-type for this study.	[5]
mc^{2}6206	Avirulent auxotrophic *M. tuberculosis* mutant (Δ*leuCD* Δ*panCD*).	[6]
AtpE(A63P)	mc^{2}6206 with a G to C SNP at position 187 bp *atpE.*	This study
Rv0678(G65fs)	mc^{2}6206 with a single nucleotide deletion at position 193 bp of *rv0678*. Resulting protein is frameshifted.	This study
Rv3006(F134S)	mc^{2}6230 with a X to X SNP at position X of Rv3006.	This study

Rv3006(R36S)	mc^{2}6230 with a X to X SNP at position X of Rv3006.	This study
Mycobacterium bovis BCG	Pasteur 1173P2 (NC_008769.1)	[7]
Plasmids		
pJLR965	CRISPRi cloning vector for *M. tuberculosis*	[8]
pCi73	pJLR965 containing sgRNA targeting *atpB* of *M. tuberculosis*	[9]
pCi7	pJLR965 containing sgRNA targeting *mmpL3* of *M. tuberculosis*	[10]
pYUB28b	Episomal expression vector for mycobacteria	[11]
pLH*cyd*	pYUB28b with *rv_1623c-rv1620c* (*cydABDC*), containing C-terminal FLAG tag on *cydB*.	[12]

Supplementary Table 2: IC_{50} values of the indicated compounds towards *M. tuberculosis* cytochrome *bd* oxidase.

Compound	IC_{50} [95% CI] (µM)	IC_{50} (fold MIC)	OCR at 1xMIC (%)
HM2-16F	21.16 [10.88-39.09]	2.11	58.86
BDQ	11.71 [4.57-27.03]	58.44	83.04
Aurachin D*	0.15 [0.091-0.27]	0.019	3.70

* The MIC of Aurachin D is determined from MIC testing against *M. smegmatis* Δ*qcr* mutant (supplementary Table 1).

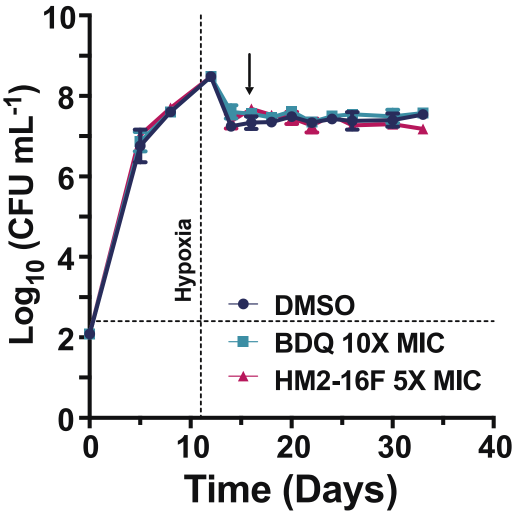

Supplementary Figure 1: Survival of *M. tuberculosis* after treatment with HM2-16F (20 μM, 5× MIC) and bedaquiline (BDQ 2 μM, 10× MIC). Hypoxia was achieved as cultures exhausted oxygen in the sealed serum vials as indicated by the decolorization of methylene blue (vertical dotted line) [12]. Compounds were added at the indicated arrow. The horizontal dotted line indicates the limit of detection for all, but the first time point, where the limit of detection was 10 CFU mL^{-1}. Error bars indicate standard deviation from n = 3 biologically independent experiments.

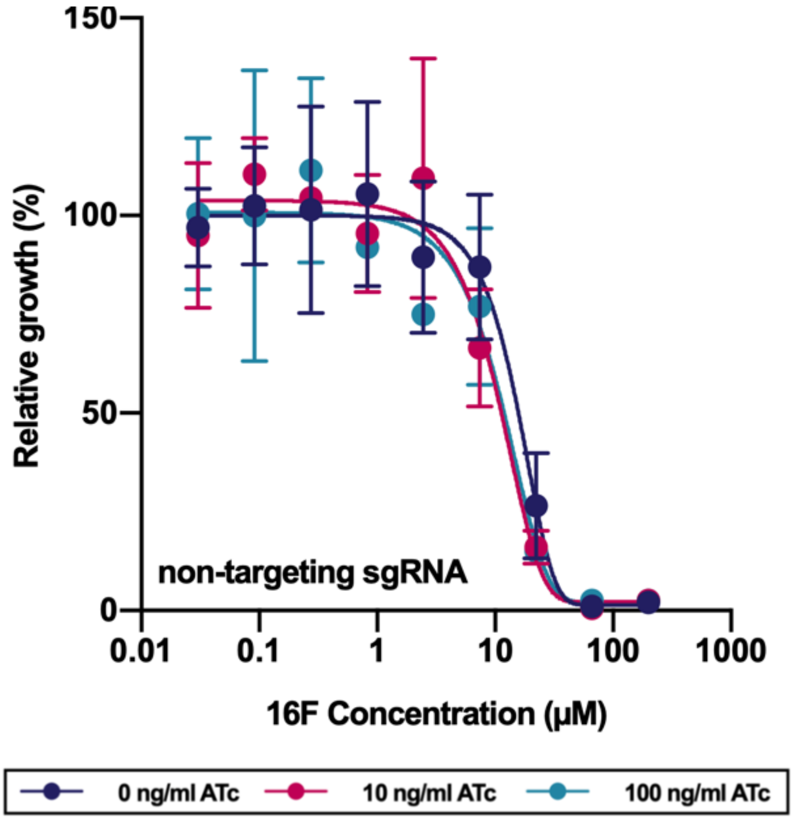

Supplementary Figure 2: Control gene knockdown with a non-targeting sgRNA (pJLR965) was induced with the indicated amounts of ATc and the MIC of HM2-16F was determined. Error bars indicate standard deviation from n = 3 biologically independent experiments.

.

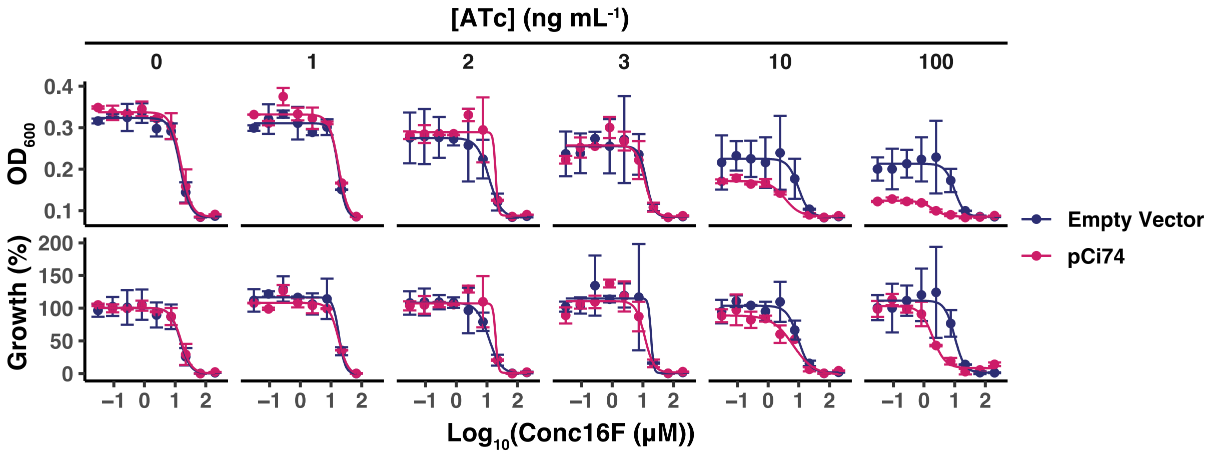

Supplementary Figure 3: Knockdown of the ATP synthase operon (*atpB* – pCi74) was induced with the indicated amounts of ATc, in the indicated strains and the growth relative to the vehicle control was determined. Raw OD_{600} absorbance values (path length = 0.33 cm) are plotted as indicated. Error bars indicate standard deviation from n = 3 biologically independent experiments.

.

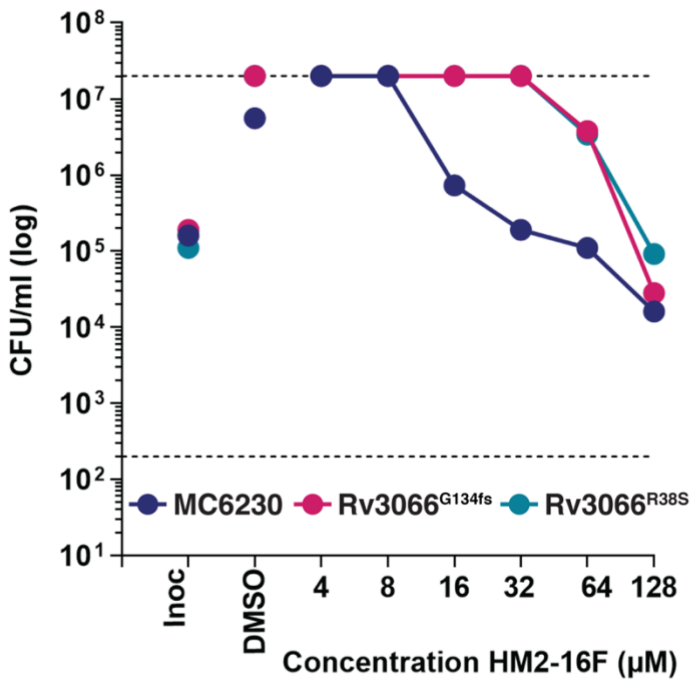

Supplementary Figure 4: Cell killing of the indicated *M. tuberculosis* strains after 10 days incubation with HM2-16F at 5x MIC. The CFU at day 0 is indicated (Inoc). MC6206 = wild-type; HM2-16-resistant mutants, transcription factor Rv3066 G134fs and R38S (Supplementary Table 1).

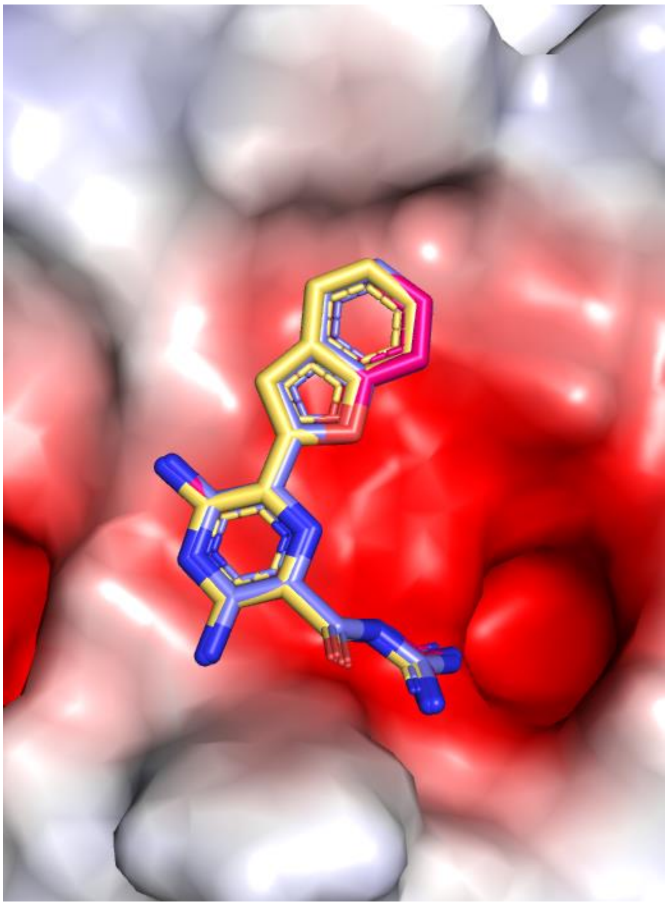

Supplementary Figure 5: The top three docked poses for HM2-16F (blue, yellow, pink) docked into the BDQ-binding site of mycobacterial F_1F_o-ATP synthase *c*-ring (PDB ID: 4V1F). The protein is shown as an electrostatic potential surface (red – electronegative, white – neutral, blue – electropositive; generated in PyMOL).

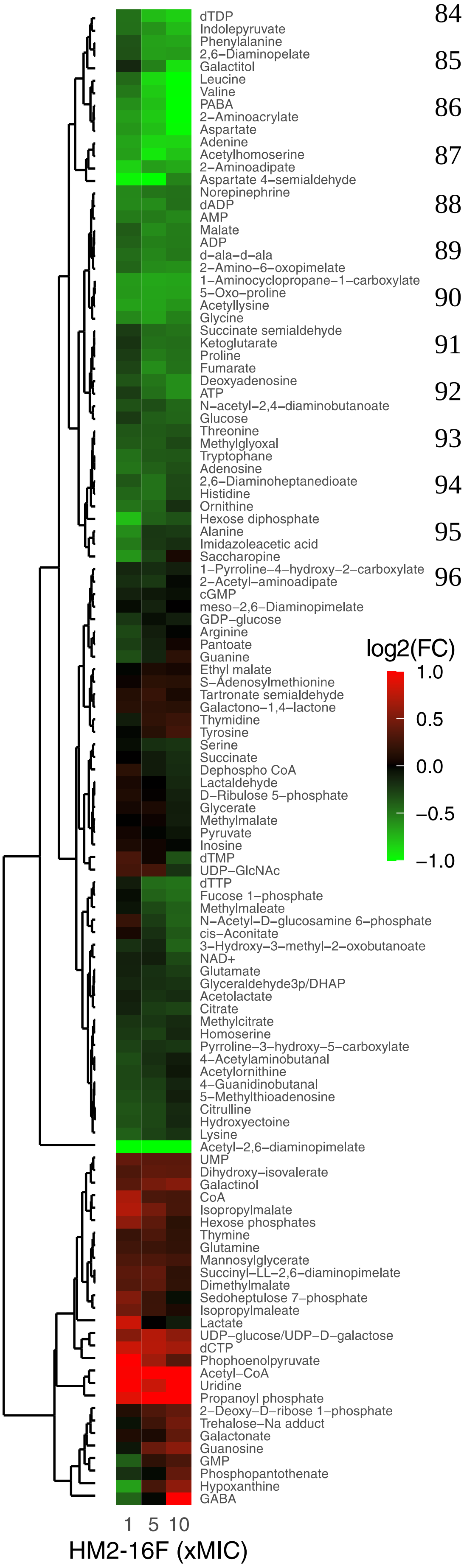

Supplementary Figure 6: Heatmap metabolite profile following 1d exposure of *M. tuberculosis* H37Rv to increasing concentrations of HM2-16F (1-10× MIC). Columns represent individual treatments as indicated. Rows denote individual metabolites measured. Hierarchical clustering was performed on the Euclidean distance matrix of this data and the resulting dendrogram and heatmap was visualized with ggplot2 in R. FC = Fold change.

Supplementary References

1. Jacob, A. E. & Hobbs, S. J. Conjugal Transfer of Plasmid-Borne Multiple Antibiotic Resistance in *Streptococcus faecalis* var. *zymogenes*. *J Bacteriol* **117**, 360–372 (1974).
2. Snapper, S. B. *et al.* Isolation and characterization of efficient plasmid transformation mutants of *Mycobacterium smegmatis*. *Mol Microbiol* **4**, 1911–1919 (1990).
3. Lu, X. *et al.* Pyrazolo[1,5-a]pyridine Inhibitor of the respiratory cytochrome *bcc* complex for the treatment of drug-resistant tuberculosis. *ACS Infect Dis* **5**, 239–249 (2019).
4. Chong SMS *et al.* Antituberculosis activity of the antimalaria cytochrome *bcc* oxidase inhibitor SCR0911. *ACS Infect Dis* **6,** 725-737 (2020).
5. Sambandamurthy, V. K. *et al.* A pantothenate auxotroph of *Mycobacterium tuberculosis* is highly attenuated and protects mice against tuberculosis. *Nat Med* **8**, 1171–1174 (2002).
6. Tufariello, J. M. *et al.* Enhanced specialized transduction using recombineering in *Mycobacterium tuberculosis*. *mBio* **5**, e01179-14 (2014).
7. Brosch, R. et al. Genome plasticity of BCG and impact on vaccine efficacy. *Proc Natl Acad Sci* **104**, 5596–5601 (2007).
8. Rock, J. M. *et al.* Programmable transcriptional repression in mycobacteria using an orthogonal CRISPR interference platform. *Nat Microbiol* **2**, 16274–16274 (2017).
9. McNeil, M. B., Ryburn, H. W. K., Harold, L. K., Tirados, J. F. & Cook, G. M. Transcriptional inhibition of the F_1F_o-type ATP synthase has bactericidal consequences on the viability of mycobacteria. *Antimicrob Agents Chemother* **64,** e00492-20 (2020).

10. McNeil, M. B. & Cook, G. M. Utilization of CRISPR interference to validate MmpL3 as a drug target in *Mycobacterium tuberculosis*. *Antimicrob Agents Chemother* **63**, e00629-19 (2019).

11. Bashiri, G., Rehan, A. M., Greenwood, D. R., Dickson, J. M. J. & Baker, E. N. Metabolic engineering of cofactor F_{420} production in *Mycobacterium smegmatis*. *PloS ONE* **5**, e15803–e15803 (2010).

12. Safarian, S. *et al.* The cryo-EM structure of the bd oxidase from *Mycobacterium tuberculosis* reveals a unique structural framework and enables rational drug design to combat tuberculosis.. *Nat Commun* **12**, 5236 (2021).

13. Berney, M., Weimar, M. R., Heikal, A. & Cook, G. M. Regulation of proline metabolism in mycobacteria and its role in carbon metabolism under hypoxia. *Mol Microbiol* **84**, 664–681 (2012).
