# Supplementary material for: An amiloride derivative is active against the F1Fo-ATP synthase and cytochrome bd oxidase of Mycobacterium tuberculosis
Source: Commun Biol. 2022 Feb 24;5:166. doi: 10.1038/s42003-022-03110-8 (PMC8873251; doi:10.1038/s42003-022-03110-8)
Supplement: Supplementary file 2 — Description of additional Supplementary Files (new) [file 42003_2022_3110_MOESM2_ESM.pdf]

Description of Additional Supplementary Files

File name: Supplementary Data 1
Description: Raw metabolomic data.

File name: Supplementary Data 2
Description: Source data for main figures.
